# Analysis of Unannotated Equine Transcripts Identified by mRNA Sequencing

**DOI:** 10.1371/journal.pone.0070125

**Published:** 2013-07-29

**Authors:** Stephen J. Coleman, Zheng Zeng, Matthew S. Hestand, Jinze Liu, James N. Macleod

**Affiliations:** 1 Maxwell H. Gluck Equine Research Center, Department of Veterinary Science, University of Kentucky, Lexington, Kentucky, United States of America; 2 Department of Computer Science, University of Kentucky, Lexington, Kentucky, United States of America; The Centre for Research and Technology, Hellas, Greece

## Abstract

Sequencing of equine mRNA (RNA-seq) identified 428 putative transcripts which do not map to any previously annotated or predicted horse genes. Most of these encode the equine homologs of known protein-coding genes described in other species, yet the potential exists to identify novel and perhaps equine-specific gene structures. A set of 36 transcripts were prioritized for further study by filtering for levels of expression (depth of RNA-seq read coverage), distance from annotated features in the equine genome, the number of putative exons, and patterns of gene expression between tissues. From these, four were selected for further investigation based on predicted open reading frames of greater than or equal to 50 amino acids and lack of detectable homology to known genes across species. Sanger sequencing of RT-PCR amplicons from additional equine samples confirmed expression and structural annotation of each transcript. Functional predictions were made by conserved domain searches. A single transcript, expressed in the cerebellum, contains a putative kruppel-associated box (KRAB) domain, suggesting a potential function associated with zinc finger proteins and transcriptional regulation. Overall levels of conserved synteny and sequence conservation across a 1MB region surrounding each transcript were approximately 73% compared to the human, canine, and bovine genomes; however, the four loci display some areas of low conservation and sequence inversion in regions that immediately flank these previously unannotated equine transcripts. Taken together, the evidence suggests that these four transcripts are likely to be equine-specific.

## Introduction

Structural and functional characteristics that define a horse are determined by the information encoded in the equine genome. Heritable aspects of equine anatomy and physiology are generated through DNA sequence variants and the differential regulation and expression of this information. Although phenotypes that define individual mammalian species largely reflect sequence differences between gene orthologs, copy number variations, and differential patterns of gene expression, some unique features may be encoded by species-specific genes. Horse-specific genes are not readily identified from available equine EST/cDNA resources due to relatively limited coverage. In addition, equine gene sets predicted *in silico* by Ensembl and NCBI will not identify horse-specific genes since they rely on homology-based projection of gene structure annotation from other species. However, the large amounts of transcriptome data generated in RNA sequencing studies almost always include reads that align to regions of the reference genome without existing gene annotation. We previously applied mRNA sequencing (RNA-seq) to eight equine tissue samples [1]. Annotation analysis characterized 19,378 transcriptional units with putative exon/intron structure. These were used to improve and refine equine gene structure annotation. However, 428 of the identified transcripts did not co-localize with any Ensembl and NCBI *in silico* gene predictions. The current project investigates these unannotated transcripts further, with detailed analysis of four that have more novel features.

## Materials and Methods

### RNA-seq Data and Unannotated Transcripts

RNA-seq (Illumina) was applied to eight equine tissue RNA samples for analysis of equine protein-coding gene structure (GEO series accession GSE 21925) [1]. Comparison of the RNA-seq derived structures with the *in silico* equine gene predictions from Ensembl (Ensembl Horse Genome Browser, database version 58.2e, May 2010; http://www.ensembl.org/Equus_caballus/; [2]) and NCBI (NCBI Equine Genome Page, March 2009; http://www.ncbi.nlm.nih.gov/projects/genome/guide/horse/) identified a subset of 428 putative protein-coding transcripts which did not overlap with any gene or pseudogene annotated by Ensembl or NCBI. The sequence for each unannotated transcript was extracted computationally from the reference genome using the start and end basepair coordinates of each individual exon. Discontiguous nucleotide megaBLAST of the non-redundant nucleotide database (NCBI BLAST Homepage; http://blast.ncbi.nlm.nih.gov/Blast.cgi; [3]) was used to make a preliminary analysis of homology. Alignments were considered significant if they had an e-value≤1e-5, a bit score≥100, and when the target sequence was annotated as a transcribed gene.

### Transcript Filtering and Selection

**Figure 1:**
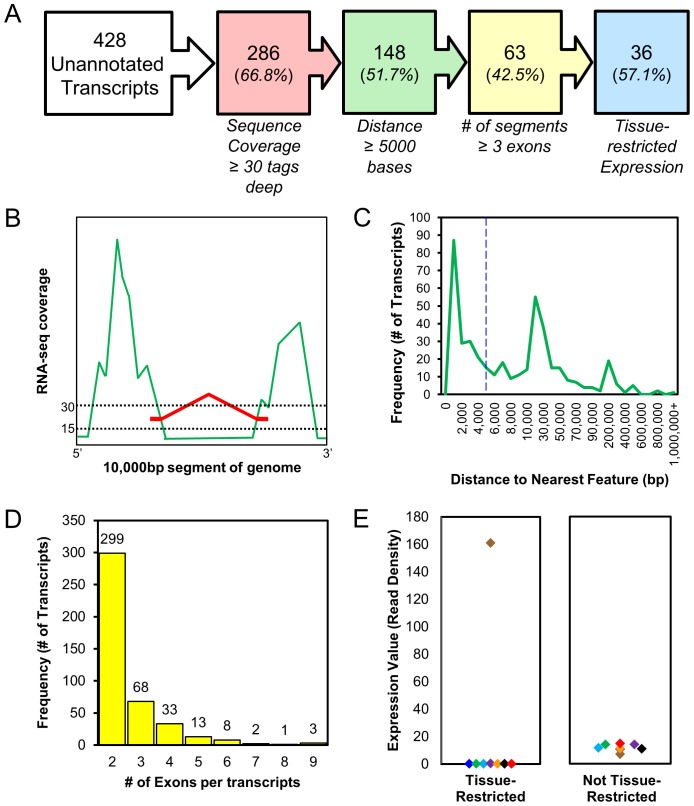
Four hundred and twenty-eight unannotated transcripts were prioritized for further study by filtering for RNA-seq read depth, distance to nearest annotated feature in the equine genome, number of putative exons, and pattern of expression. The filters were applied iteratively leading to a prioritized set of 36 transcripts (A). The depth of coverage threshold was 30 reads [the green peaks represent depth of exon coverage and the red line represents depth of splice junction coverage] (B). The distance to nearest feature threshold was set at 5,000 bp (C). Transcripts were selected if they had 3 or more putative exons (D). Transcripts were selected if they displayed a tissue-restricted pattern of expression as determined by the extreme studentized deviate or Grubb’s test (E). Each data point on the graphs represents a measurement of the same transcript in a different tissue sample.

The unannotated transcripts were prioritized for analysis through the application of four filters (Figure 1A). First, the transcripts were filtered for a sequence coverage depth threshold of 30 reads. This minimum depth was twice the level required for gene structure annotation [1] and was selected to ensure coverage across the full length of each exon and represented a level of expression sufficient to capture sequence reads defining splice junctions where they existed (Figure 1B). Second, a distance threshold of 5,000 or more bases from the nearest annotated equine gene was applied. This minimum distance reduced the likelihood that the selected unannotated transcripts would represent additional exons of previously known equine genes. The distance distribution for all unannotated transcripts in the dataset was trimodal in appearance with peaks at approximately 1,000, 20,000, and 200,000 bases (Figure 1C). The large single peak at 1,000 bases reached its minimum frequency at 5,000 bases. Third, a threshold of three or more exons was applied in order to prioritize longer transcripts with structural features consistent with multi-exon mRNA (Figure 1D). It was also noted that a much higher proportion of the two exon transcripts as compared to the longer multi-exon transcripts represented additional exons to previously annotated genes. Finally, transcripts were filtered based on their pattern of expression across the eight equine tissue samples. Transcripts were prioritized if they had a tissue-restricted pattern of expression within the current data set, which was identified by application of the extreme studentized deviate or Grubb’s outlier detection test [4], [5]. For each unannotated transcript, Z-scores using the normalized expression values determined in the 8 individual tissue samples. If the Z-score for a particular tissue was greater than the critical value of Z = 2.13, it was considered to be significantly deviated from the mean of all measurements and indicated a tissue-restricted expression pattern (Figure 1E).

Transcript selections for more detailed analysis were also based on the calculated open reading frames (ORFs) and re-evaluation of homology across species. The assembled nucleotide sequence for each prioritized transcript was translated into six amino acid sequences (one for each ORF) using the ExPASy Translate tool (http://web.expasy.org/translate/; [6]). For each of the six open reading frames, the longest calculated peptide sequence was identified. Transcripts were only considered for further analysis if at least one of the ORFs predicted a peptide sequence 50 amino acids or longer. This threshold was selected because it is more than twice the length expected to be generated by chance and most primary products of translation are 50 amino acids or longer [7]. BLASTX analysis [8] was used to query the unannotated transcripts against the non-redundant protein database (NCBI BLAST Homepage; http://blast.ncbi.nlm.nih.gov/Blast.cgi). Alignments were considered significant if they had an e-value≤1e-5 and a bit score≥100. Transcripts were selected for further analyses if they had no demonstrated homology to known genes. To confirm a lack of homology, the transcripts were mapped to the equine reference genome (EquCab2; [9]) by BLAT [10] using default parameters. The identified locus was used to search the UCSC comparative mapping tracks [11]. Structural annotation for the selected transcripts was refined using additional 100 bp Illumina sequence reads (GEO series accession GSE 46858, NIH Sequence Read Archive accession SRP022567) generated from the same original tissue RNA samples [1]. Full analysis details of these sequence reads are presented as supplemental methods (Methods S1).

### Expression Validation

Expression levels of the selected unannotated transcripts were validated by RT-PCR assays. Primer pairs were selected using the Primer3 design tool [12] and targeted to have an optimal melting temperature of 60°C ^+^/−3°C with an optimal length of 20 bp ^+^/−2 bp, and produce an amplicon that spanned all exons in the selected transcript. Predicted primer specificity was assessed by *in silico* PCR (UCSC Genome Browser; http://genome.ucsc.edu/cgi-bin/hgPcr?command=start) using default matching parameters and a maximum product size equal to the genomic interval of the corresponding transcript. Specificity was accepted if the primers mapped only to loci that overlapped the genomic interval of the targeted transcript of interest. Selected primers are presented in Table 1.

**Table 1:** Selected primer sequences for expression and structure validation.

Unannotated Transcript	Primer	Sequence	Length (bp)	T_m_ (°C)
UU18303	F	GAAGGAGAGCAGAGCTTGGA	20	59.83
UU18303	R	GAAGGAGAGCAGAGCTTGGA	20	59.84
UU12205	F1	AGGAGTGTGCATCCCACTTT	20	59.58
UU12205	F2	TGAGAAGGAAGCCAAGGAAA	20	59.93
UU12205	R1	TAATGCCTGGCCTATGGAAG	20	60.05
UU12205	R2	TTGTTACTGTGCGAACTCTGC	21	59.14
UU18376	F1	TGTGAAGGAGGTGAACTGGA	20	59.23
UU18376	F2	AACTGCCCAAGTCACACAGTT	21	59.68
UU18376	R1	GAATTTGCTTCTGTGCGTTG	20	59.47
UU18376	R2	AACTGTGTGACTTGGGCAGTT	21	59.68
UU18376	R3	ACTTGCCCTCTCTCGGTCTT	20	60.39
UU1814	F	TTTGTACAGGGCCCTTTGTG	20	60.91
UU1814	R	GCAGTCTCTTCACCCAGCTC	20	60.14
RPLP0	F	CTTCATTGTGGGAGCAGACA	20	59.83
RPLP0	R	GCCTTGACCTTTTCAGCAAG	20	59.99

Total RNA from equine testes and cerebellum were reverse-transcribed into cDNA using the Promega Reverse Transcription System reagents and protocol (Promega Corporation, Madison, WI, Cat# A3500) and an equal ratio of oligo-dT and random hexamer primers. PCR was performed on an Eppendorf Mastercycler Gradient thermocycler (Eppendorf, Hanover, Germany) using AmpliTaq Gold 360 PCR master mix (Applied Biosystems, Foster City, CA, Cat# 4398881). PCR products were visualized using the DNA1000 analysis kit on an Agilent 2100 Bioanalyzer (Agilent Technologies, Inc., Santa Clara, CA; Cat# 5067-1504). Each primer pair was tested against testes cDNA and cerebellum cDNA, with minus RT, genomic DNA, and water as negative controls.

### Structural Validation

Transcript structure was validated by Sanger sequencing of the PCR amplicons using the ABI Big Dye Terminator v3.1 Cycle Sequencing Kit (Applied Biosystems, Foster City, CA, Cat# 4337455). Briefly, 10 to 20 ng of the PCR product was combined with Big Dye master mix (Big Dye v3.1 ready reaction mix, 5X Big Dye sequencing buffer, 5 µM forward or reverse primer, and deionized water). The sequencing reaction was performed for 25 cycles using the same primers as for the initial PCR amplification in a total reaction volume of 10 µl. Reaction products were ethanol precipitated, combined with 30 µl of HiDi formamide and visualized on an ABI3730xl DNA Analyzer (Applied Biosystems; Foster City, CA). Resulting sequence data were compared with the original RNA-seq assembled sequence contig for each unannotated transcript using BLAT (Kent 2002).

### Sequence and Synteny Conservation

Genomic regions surrounding each unannotated transcript were analyzed for synteny and sequence conservation. Predicted and known genes surrounding each transcript were identified in an approximately 1MB interval. Interval size was selected based on the objective of anchoring both ends of the genomic interval with an annotated equine gene. Synteny was evaluated by identifying orthologs in the human, dog, and cattle genomes using the relationships established by EnsemblCompara [13]. For sequence comparisons, the genomic sequences for horse, human, dog, and cattle were extracted using Ensembl BioMart [14] for the regions surrounding each unannotated transcript defined on either side by the closest gene models with orthologs identified across all four species. The equine sequence was compared to each of the other species independently using BLASTZ with default single coverage parameters and dot plots showing the comparison of the sequences generated by Advanced PipMaker [15].

### Functional Predictions

Functional properties for predicted proteins encoded by the unannotated transcripts were made by conserved domain searches and detection of subcellular localization signal sequences. The amino acid sequences predicted by all six ORFs for each unannotated transcript were assembled into a combined FASTA file. Conserved domains were identified by comparison against the NCBI Conserved Domain Database [16], [17], [18]. Identified domains were considered significant below an e-value threshold of 1e-5. Subcellular localization signals were identified using the TargetP 1.1 analysis server (Center for Biological Sequence Analysis at DTU, http://www.cbs.dtu.dk/services/TargetP/). This tool makes a location assignment based on the predicted presence of N-terminal signal peptide or mitochondrial targeting peptide presequences. Predictions were assigned a reliability class (RC) value of 1 to 5 indicating their relative strengths, with lower values associated to stronger predictions [19]. Subcellular localizations were only considered for predictions with an RC value of 1 or 2.

## Results

### Unannotated Transcripts

**Figure 2:**
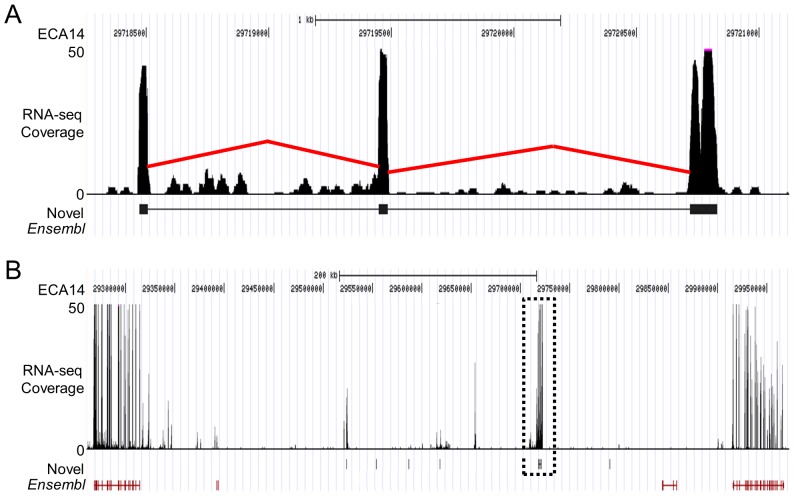
Example of an unannotated equine transcript. The upper panel shows approximately 3KB of ECA14 containing a single unannotated transcript (A). The black peaks represent depth of coverage by the RNA-seq reads and the red lines represent putative splice junctions identified by MapSplice [20]. The gene model immediately below is the annotation for this transcript derived from the RNA-seq data. The lower panel shows a 700 KB region of ECA14 surrounding the transcript (dotted box outline) illustrating that there is no annotated gene or *in silico* gene prediction overlapping this genomic interval (B). The nearest RNA-seq data not included in the transcript model is approximately 60 KB away and the nearest gene prediction is nearly 120 KB away.

**Figure 3:**
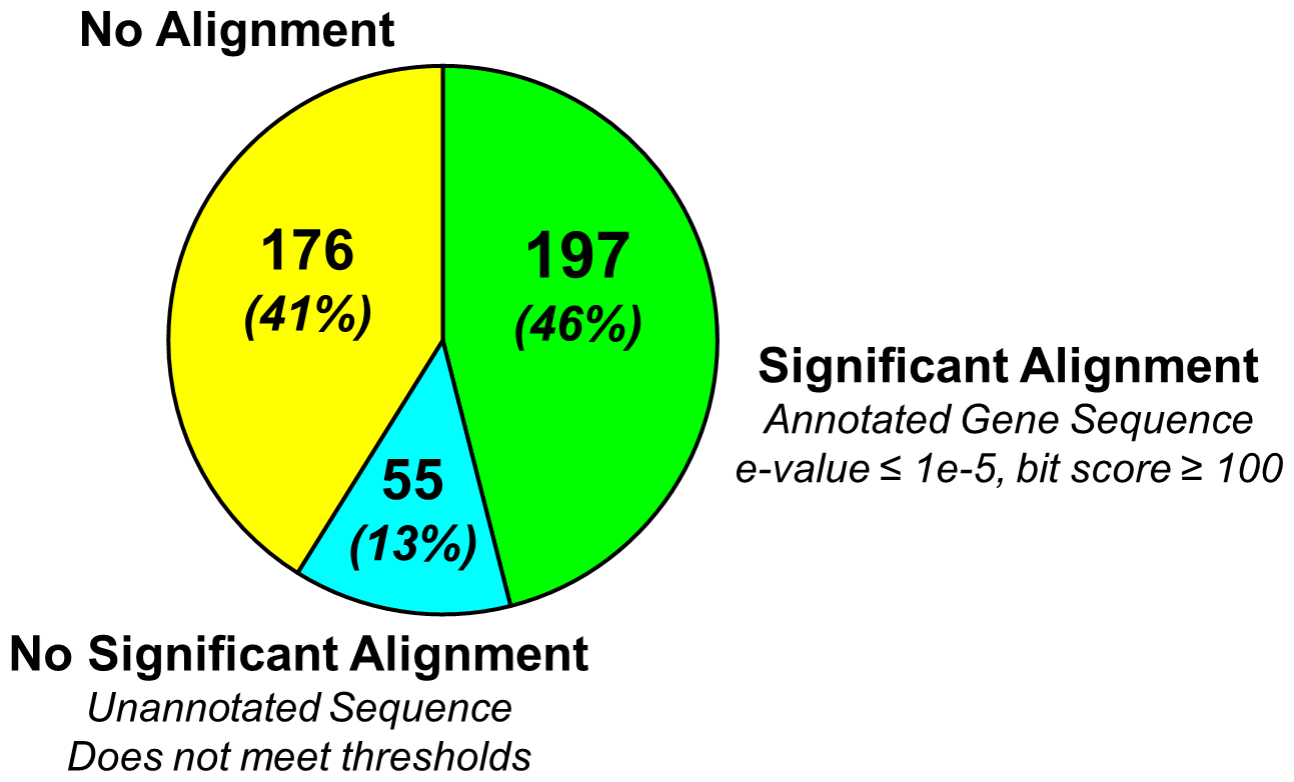
Analysis of homology by discontiguous megaBLAST. One hundred and ninety-seven (46%) of the unannotated equine transcripts aligned to sequences annotated as genes in other species, 55 (13%) aligned to unannotated sequences or below significance threshold, and the remaining 176 (41%) generated no alignments at all.

The 428 unannotated equine transcripts contained 2–9 exons (median = 2), were an average of 643 basepairs in length (range 74 to 10,401), and had an average RNA-seq coverage depth of 77 (range 19 to 3,042). A representative example is shown in Figure 2. Discontiguous megaBLAST (e-value≤1e-5; bit score≥100; associated gene annotation) was used to query the non-redundant nucleotide database as a preliminary analysis of homology. One hundred and ninety-seven (46%) aligned to annotated gene sequences in other species, 55 (13%) aligned to unannotated sequences or below threshold, and the remaining 176 (41%) generated no alignments at all (Figure 3). The transcript sequence assembly data have been deposited at DDBJ/EMBL/GenBank under the accession GAJF00000000. The version described in this paper is the first version, GAJF01000000.

### Transcript Structure Validation

**Figure 4:**
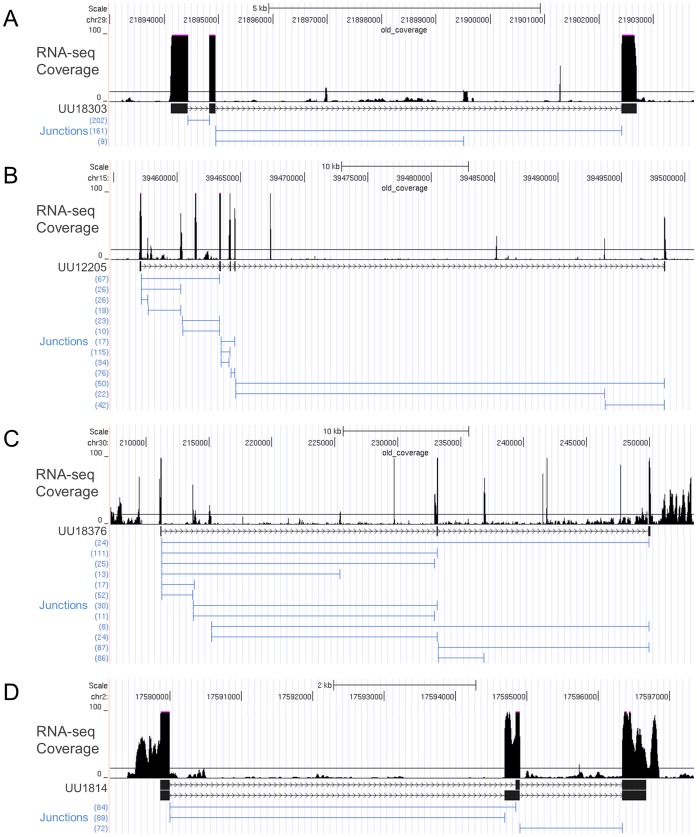
Structural details for 4 selected unannotated equine transcripts. The black peaks represent RNA-seq read depth and the blue lines represent putative splice junctions identified by MapSplice [20]. Support for the splice junctions (i.e. number of aligned reads that cross the junction) are located on the left side of each figure. Immediately below the exon coverage peaks are predicted structural models for the major transcripts. RNA-seq analysis details are included as supplemental methods. UU18303 (A), UU12205 (B), UU18376 (C), and UU1814 (D).

Thirty-six transcripts (Table 2) were prioritized for further study base on filtering criteria defined in the Materials and Methods section. All 36 of these supported at least one open reading frame (ORF) predicting a translated protein of 50 amino acids or more. Four transcripts (IDs = UU18303, UU12205, UU18376, and UU1814) were analyzed further to validate sequence identity and structure. Based on RNA-seq read alignments, putative alternative splicing patterns were observed for each transcript (Figure 4). Splice junction results for transcript UU18303 indicated the presence of a highly expressed major transcript combined with a shorter and very low expressed minor transcript terminating in an alternate exon. Similarly, two alternate mRNA variants, differing in size by 153 basepairs, were identified for UU1814. They result from alternative splice acceptor/donor usage in the middle exon. Major isoforms could not be resolved for transcripts UU12205 and UU18376. In both instances, the reported splice junctions supported multiple pathways through the exons.

**Table 2:** Description of selected transcripts.

UnannotatedTranscript	Locus	Number ofExons	Length(bp)	Homolog (similar to)
UU537	ECA1: 70810673-70814839	6	654	FXYD4 (*Sus scrofa*)
UU759	ECA1: 93611754-93614921	4	494	N/A
UU976	ECA1: 120923885-120927741	5	1397	TMEM202-like (*Bos taurus*)
UU1097	ECA1: 129776131-129787516	3	421	N/A
UU1304	ECA1: 147378438-147392725	5	5976	hypothetical protein (*Pan paniscus*)
UU1795	ECA2: 15281173-15286110	3	338	N/A
UU1814	ECA2: 17,589,860-17,596,668	3	643	N/A
UU4133	ECA4: 94369944-94374962	3	380	N/A
UU4544	ECA5: 27060113-27081749	4	405	C1orf97 (*Pan paniscus*)
UU5985	ECA7: 20765366-20771304	3	1486	C11orf34 (*Pan paniscus*)
UU6001	ECA7: 24780964-24782435	3	362	N/A
UU6079	ECA7: 29491656-29501879	3	1713	uncharacterized transcript (*Pongo abelii*)
UU6650	ECA7: 90169710-90176775	4	492	SVIP (*Canis lupus familiaris*)
UU6651	ECA7: 90187426-90198224	4	422	uncharacterized transcript (*Canis lupus familiaris*)
UU6720	ECA8: 3386523-3389899	4	514	hypothetical protein (*Ailuropoda melanoleuca*)
UU7648	ECA9: 37795498-37818967	4	282	hypothetical protein (*Papio anubis*)
UU10179	ECA12: 20494123-20515958	4	364	MS4A (*Bos taurus*)
UU11376	ECA14: 22138968-22142919	3	348	TIMD4 (*Canis lupus familiaris*)
UU11445	ECA14: 29718473-29720828	3	182	LOC100335778 (*Bos taurus*)
UU11917	ECA15: 5089445-5092230	3	335	N/A
UU12205	ECA15: 39,457,033-39,498,454	5	392	N/A
UU13082	ECA16: 41994906-42002761	3	1465	hypothetical protein (*Sus scrofa*)
UU13779	ECA17: 80469119-80479703	8	869	1700029H14Rik (*Mus musculus*)
UU14104	ECA18: 67071149-67074077	5	793	PRSS58-like (*Canis lupus familiaris*)
UU15940	ECA22: 34285501-34286605	3	389	WFDC15B (*Bos taurus*)
UU16194	ECA23: 19006045-19011880	3	1093	C9orf57 (*Sus scrofa*)
UU16781	ECA24: 42686132-42721148	9	10410	MEG3 (*Homo sapiens*)
UU16784	ECA24: 43528445-43546083	3	1142	uncharacterized transcript (*Canis lupus familiaris*)
UU17491	ECA26: 37127724-37141004	4	820	N/A
UU17605	ECA27: 3312336-3325363	4	829	N/A
UU17720	ECA27: 23956623-23966552	3	1124	N/A
UU17987	ECA28: 34400567-34404183	3	291	KCTD17 (*Papio anubis*)
UU18303	ECA29: 21,894,122-21,902,694	3	692	N/A
UU18376	ECA30: 211,164-253,935	6	747	N/A
UU19050	ECAX: 54336811-54337514	3	302	uncharacterized transcript (*Pan troglodytes*)
UU19054	ECAX: 55430384-55505951	7	2334	N/A

**Figure 5:**
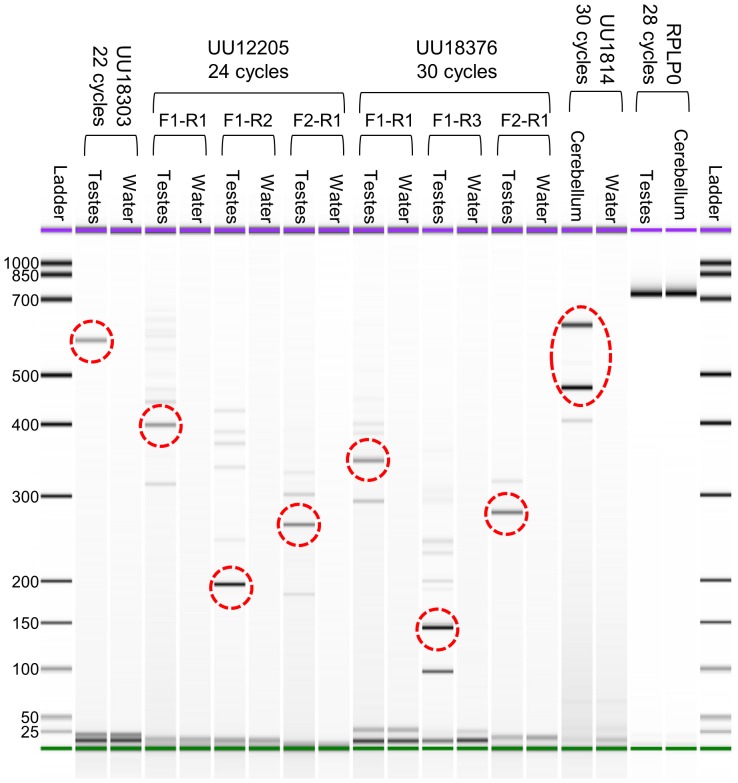
Validation of expression for unannotated transcripts UU18303, UU12205, UU18376, and UU1814. RT-PCR was used to amplify cDNA from each transcript followed by visualization on an Agilent Bioanalyzer 2100. RPLP0 was included as a positive control for both testes and cerebellum template cDNA. Water was used as a negative control. Transcripts UU12205 and UU18376 required a second round of amplification with nested primers to resolve an amplicon for direct sequencing. Individual transcripts with primer pair, template, and number of cycles used are displayed across the top. The expected products are circled in red.

Expression and structural annotation for the selected transcripts were validated by RT-PCR and Sanger sequencing, which confirmed expression in targeted tissue for all the selected transcripts (Figure 5). PCR amplicons generated with the initial primer sets were of the predicted size for the major transcripts of UU18303 and UU1814. However, internal alternative splicing structures for UU12205 and UU18376 necessitated the design of nested primers to resolve a single band suitable for Sanger sequencing. Nucleotide sequencing data confirmed the identity of each transcript.

### Sequence and Synteny Conservation

**Figure 6:**
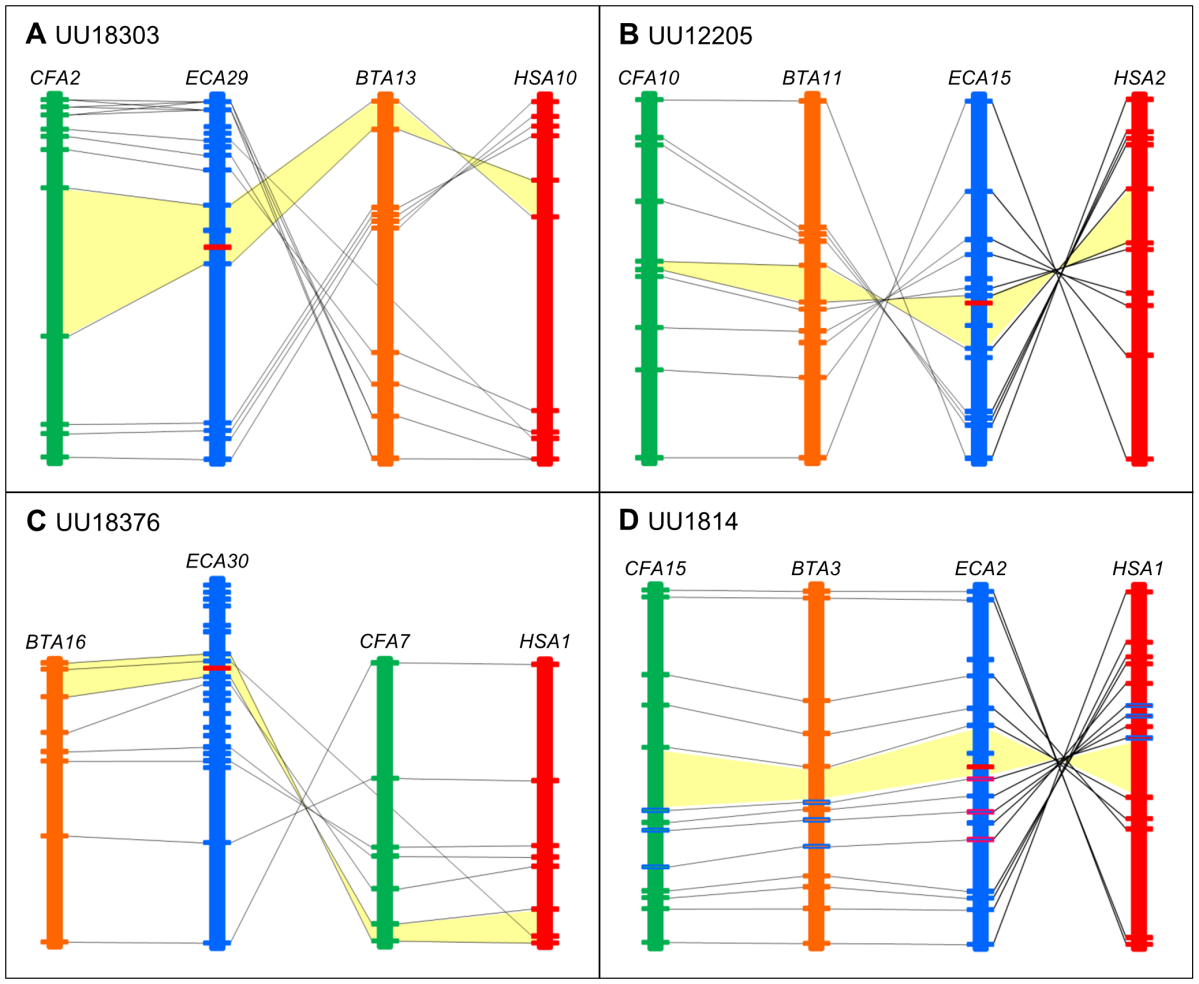
Conserved synteny of genomic regions of the four unannotated equine transcripts and the corresponding regions in the human, canine, and bovine genomes. The unannotated transcripts [UU18303 (A), UU12205 (B), UU18376 (C), and UU1814 (D)] are highlighted on each diagram as a solid red horizontal bar. The diagram for UU1814 includes additional highlighted genes (in red on ECA2, blue on other species) on ECA2. These highlighted genes represent a cluster of conserved KRAB-ZFP to which UU1814 could be related.

**Figure 7:**
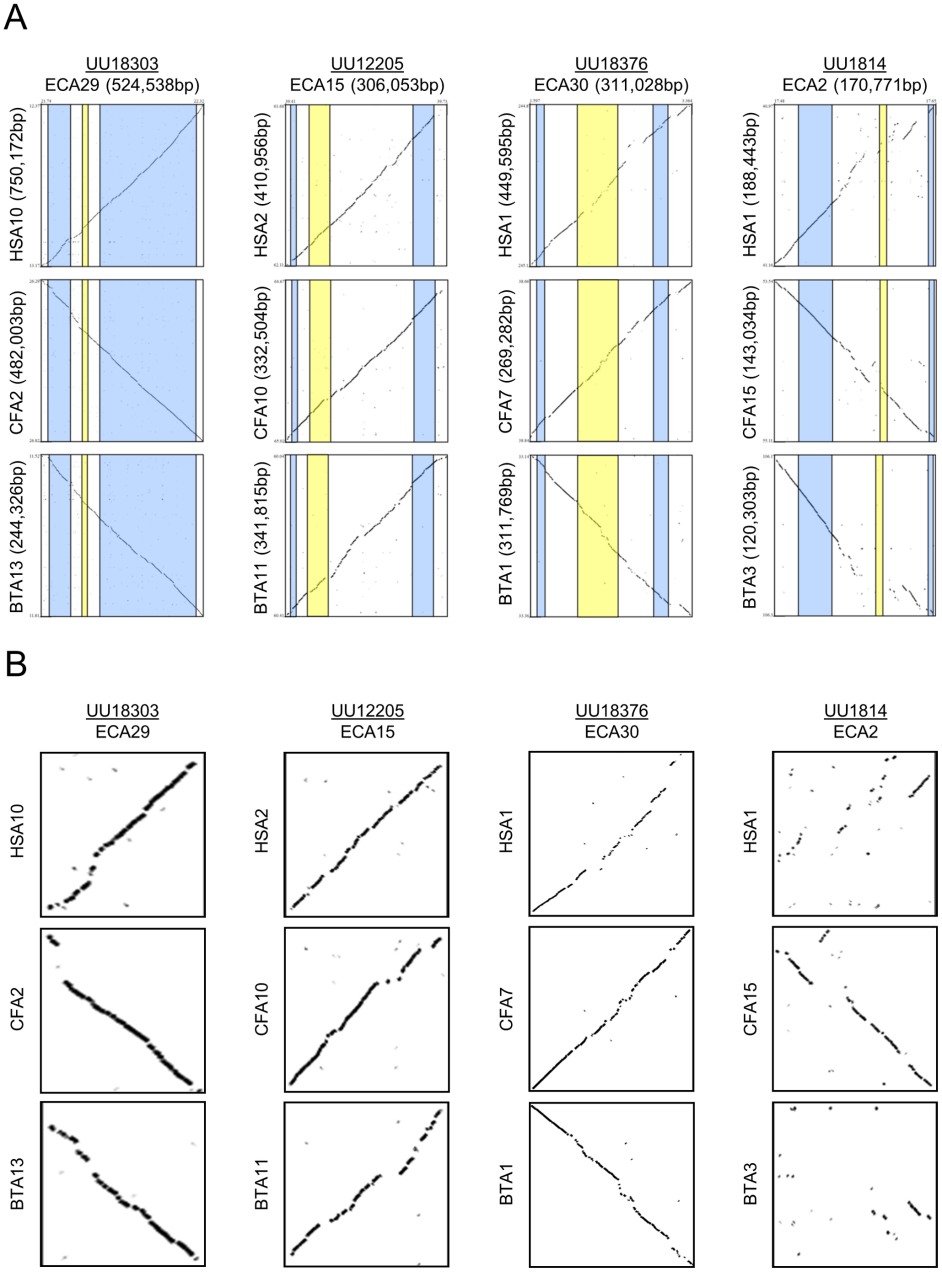
Dot plots depicting sequence comparisons between the genomic regions of the four unannotated equine transcripts and the corresponding regions in the human, canine, and bovine genomes (A). The genomic intervals of the unannotated transcripts are highlighted in yellow and the nearest conserved Ensembl protein-coding gene prediction in the flanking regions are highlighted in blue. Dot plots depicting sequence comparisons between the specific interval of the unannotated equine transcripts (yellow segment in panel A) and the corresponding regions in the human, canine, and bovine genomes (B).

For each transcript, the ∼1 MB intervals selected to assess synteny and sequence conservation contained between 19 and 25 predicted genes. Levels of conserved synteny (homologous gene pairs) ranged from 40 to 75 percent (Figure 6). In all cases, the gene order showed an inversion relative to the human genome extending beyond the interval in question. Inversions relative to the equine genome were also present in canine and bovine for UU12205 and in canine for UU18376. Two transcripts (UU18303 and UU1814) had adjacent gene predictions for which no ortholog was identified in human, cow, or dog. Predicted equine genes immediately adjacent to UU12205 had no human or cow ortholog and the orthologs detected in dog mapped to chromosomes outside the region of conserved synteny. Transcript UU18376 had genes with orthologs on both sides, but came from the interval with the lowest overall conserved synteny (40%). Overall sequence conservation for the entire intervals was approximately 73% across all four regions. Transcript UU1814 demonstrated particularly poor conservation with human and cattle across the interval defined by the transcript. Dot plots showing the sequence-based comparisons of the horse intervals with those from human, dog, and cattle are shown in Figure 7.

### Functional Predictions

Functional predictions were investigated for the four transcripts. Conserved domain search with translated peptide sequence for transcript UU1814 identified a kruppel associate box (KRAB) domain in the longest ORF at the same relative location in two isoforms predicted by alternative splicing (expect values of 2.48e-21 and 3.55e-22 respectively). Similarly, a mitochondrial targeting peptide (mTP) sequence was predicted (reliability class = 1) in both isoforms, also in the fourth ORF. The remaining transcripts did not generate any functional domain predictions.

## Discussion

Previously described RNA-seq data from eight equine tissue mRNA samples [1] identified 428 putative transcripts not previously described in the horse. Initial BLAST analysis indicated significant similarity with annotated genes from other species for 197 of them, suggesting that these sequences represent the identification (either in whole or in part) of orthologous genes not currently included in the equine protein-coding gene set (http://www.ensembl.org/Equus_caballus/). In many cases, these equine orthologs could have been identified by the automated genome annotation pipelines, but were potentially omitted from the final gene set because they failed quality thresholds due to the lack of experimentally derived expression data. For some of the transcripts, lower levels of the nucleotide identity and percent coverage in the BLAST alignment suggest that they may represent a conserved domain instead of shared gene identity. The subset of 176 transcripts that did not yield a BLAST alignment have the potential to represent novel equine gene structures, but will require further analysis as illustrated by the 4 examples in the current study.

Comparison of the predicted amino acid sequence generated from transcript UU1814 against the conserved domain database identified a kruppel associated box (KRAB) domain. KRAB domains are associated with zinc finger proteins (ZFP). KRAB-ZFPs constitute the largest individual family of transcription factors in mammals. The KRAB domain functions as a transcriptional repressor, associating with accessory proteins to prevent assembly of the transcriptional machinery [21]. This class of transcription factors has demonstrated significant expansion throughout evolution through gene duplication [22] with their functional roles associated with mammalian speciation [23]. Conservation of the zinc finger motifs in these genes after duplication is low [22]. This flexibility contributes to the adaptability of zinc finger proteins, the rapid expansion of this gene family, and suggests an explanation as to why no zinc finger motifs (C2H2 domains) were identified in UU1814. An alternative explanation is that the zinc finger motifs were lost after the introduction of a stop codon or point mutation, which has been shown to occur frequently [22]. KRAB-ZFP tend to be organized as gene clusters [23], [24], owing to their pattern of expansion by gene duplication and as a mechanism to enhance expression. Interestingly, UU1814 is located on ECA2 immediately upstream from 3 predicted Ensembl genes, all of which contain a KRAB and C2H2 zinc finger domains (panel D, Figure 6). BLASTN analysis of UU1814 against these three genes does not identify sequence similarity outside of the KRAB domain. Subcellular localization of UU1814 by TargetP [19] predicts a mitochondrial targeting peptide (mTP), suggesting that it could play a functional role in regulating the transcription of mitochondrial encoded genes. Directed functional analysis will be required to confirm if these predictions are accurate. The potential exists that UU1814 describes an equine-specific transcriptional repressor with cerebellum-restricted expression.

The functional predictions for UU1814 suggest small scale gene duplication as the probable mechanism of its origination. Synteny and sequence conservation of the regions surrounding each unannotated transcript were analyzed in an effort to suggest likely mechanisms of origination for the other transcripts. While sequence conservation was generally high (73% across the regions surrounding each transcript), these analyses identified areas of low conservation immediately adjacent to the transcripts. This was primarily in terms of synteny, where the nearest one or two predicted loci (either upstream or downstream on the chromosome) did not show conservation across the human, canine, and bovine genomes. It was also apparent from the sequence comparisons that the regions defined by the selected synteny groups were of different overall lengths between species. Taken together with the synteny differences, these results could indicate that like UU1814, the remaining 3 unannotated transcripts arose through small scale duplication events. Alternatively, it is possible that these transcripts represent genes which have already been lost from other mammalian genomes and are in the process of being removed from the equine genome by pseudogenization.

Several questions remain regarding these four unannotated transcripts. First and foremost is whether they are expressed in any other species. While BLAST analysis failed to identify orthologous genes, the level of sequence conservation across each locus suggests the possibility that they may exist in other species and have not yet been identified. Evaluation of expression across other equids, perrisodactyls, and other mammals will help to determine if these transcripts are indeed equine-specific. Additionally, it will be important to determine if these transcripts are translated, the functional role for the encoded protein, and how it is regulated. Detailed studies of the transcriptome by next generation sequencing technologies will continue to identify novel mRNAs with the potential to provide new insight into phylogenetic relationships and speciation.

## Supporting Information

Methods S1 (DOCX)
