# Correction: Analysis of Unannotated Equine Transcripts Identified by mRNA Sequencing

**DOI:** 10.1371/annotation/b9b4a26a-4eb1-482f-b99d-e248f8ca31fa

**Published:** 2013-09-19

**Authors:** Stephen J. Coleman, Zheng Zeng, Matthew S. Hestand, Jinze Liu, James N. Macleod

A funding organization and a preferred acronym for one of the funding organizations already listed were incorrectly omitted from the Funding Statement. The Funding Statement should read: "This work was funded by the University of Kentucky Gluck Equine Research Foundation, the Morris Animal Foundation, the Lourie Foundation, the National Science Foundation (EF-0850237), and the Kentucky Biomedical Research Infrastructure Network (KBRIN) supported in part by National Institutes of Health grant KY-INBRE P20 RR16481. The funders had no role in study design, data collection and analysis, decision to publish, or preparation of the manuscript." 

