# Supplementary material for: Analysis of Unannotated Equine Transcripts Identified by mRNA Sequencing
Source: PLoS One. 2013 Jul 29;8(7):e70125. doi: 10.1371/journal.pone.0070125 (PMC3726457; doi:10.1371/journal.pone.0070125)
Supplement: Methods S1 — (DOCX) [file pone.0070125.s001.docx]

**Supplemental Methods**

RNA isolation and library preparation was performed as previously described [1]. Briefly, total RNA was isolated from eight equine tissue samples (articular cartilage, synovial membrane, placental villous, testes, cerebellum, 34-day embryo, LPS-stimulated articular cartilage, and LPS-stimulated synovial membrane) by various techniques. Library preparation was according to Illumina’s standard mRNA-seq kit protocol (Transcriptome Analysis: mRNA-seq, http://www.illumina.com/pages.ilmn?D = 291) and sequencing performed in 100 cycles (generating 100bp sequence reads).

The 35bp sequence reads generated previously from the same RNA samples [1] were combined with the 100bp sequence reads for structural annotation analysis. Prior to mapping, sequence reads were trimmed based on quality score, with N nucleotides and sequencing linkers removed using custom scripts (based on GAPSS [2]). They were subsequently mapped to the equine reference genome (EquCab2), [3] using MapSplice [4]. Reads which remained 45bp or greater after trimming were used to identify splice junctions. Junctions were filtered for a combined average read mismatch of less than or equal to 1, entropy greater than or equal to 2, and intron length greater than or equal to 50bp and less than or equal to 200,000bp. Sequence reads 44bp and shorter were used to define exon structures.

**References**

1. Coleman SJ, Zeng Z, Wang K, Luo S, Khrebtukova I, et al. (2010) Structural annotation of equine protein-coding genes determined by mRNA sequencing. *Animal Genetics* 41(Suppl. 2): 121-130.
2. Hestand MS, Klingenhoff A, Scherf M, Ariyurek Y, Ramos Y et al. (2010) Tissue-specific transcript annotation and expression profiling with complementary next-generation sequencing technologies. *Nucleic Acids Research*. 38(16):e165.
3. Wade CM, Guilotto E, Sigurdsson S, Imsland F, Lear TL, et al. (2009) Genome sequence, comparative analysis, and population genetics of the domestic horse (*Equus caballus*). *Science* 326(5954): 865-867.
4. Wang K, Singh D, Zeng Z, Coleman SJ, Huang Y, et al. (2010) MapSplice: Accurate mapping of RNA-seq reads for splice junction discovery. *Nucleic Acids Research* 38(18): e178.
